# PROTOCOL: Education and Covid‐19: An evidence and gap map

**DOI:** 10.1002/cl2.1318

**Published:** 2023-03-19

**Authors:** Sarah Miller, Ciara Keenan, Erin Early, Karen McConnell, Leonor Rodriguez

**Affiliations:** ^1^ School of Social Sciences, Education and Social Work Queen's University Belfast Belfast UK; ^2^ Campbell UK & Ireland Queen's University Belfast Belfast UK; ^3^ Institute of Education University College London London England; ^4^ Ulster University Newtownabbey UK; ^5^ UNESCO Child and Family Research Centre NUI Galway Galway Ireland

## Abstract

This is the protocol for a Campbell evidence and gap map. The objectives are as follows: identify and map all existing primary studies, systematic reviews (published and unpublished), guidelines and policies on education during the Covid‐19 pandemic, creating a live, searchable and publicly available evidence and gap map.

## 1 BACKGROUND

### 1.1 The problem, condition or issue

The Covid‐19 pandemic has resulted in global restrictions and lockdowns, not least of which has been the periodic and prolonged closure of schools. The impact of these public health measures on children and young people is, and will continue to be, both wide‐ and long‐ranging, and will not be fully understood for many years to come (Newlove‐Delgado et al., 2021). From the start of 2020 teaching and learning changed considerably for pupils and teachers as classrooms moved from school to the home (Richmond et al., 2020). Many statutory exams were cancelled, and technology and parents began to play a major and unprecedented role in children's learning. There is now considerable concern and uncertainty for children and young people with respect to their educational outcomes and trajectories, particularly for those who are at disproportionate risk of poor outcomes (e.g. children from disadvantaged backgrounds) (Weidmann et al., 2021). Projections and data simulations suggest that three months of school closures could result in up to a year of ‘learning loss’ (Kaffenberger, 2021; Kuhfeld et al., 2020), however, it is also considered that remediation will play an important role in ensuring that potential adverse impacts are minimised (Azevedo et al., 2021). Research has also highlighted that remote learning was positive for some students who may have struggled in traditional school environments by enabling an individualised pace of learning, and resulting in higher self‐efficacy, competence and persistence. Time spent at home may have encouraged young people to discover new hobbies and talents (art, music) which may have also given them a sense of meaning and control (Dvorsky et al., 2020). To this end governments have responded to this shock to education by instigating initiatives to support schools, teachers and pupils. In the UK for example, this has been through recovery premium payments to schools and the rollout of the National Tutoring Programme, summer schools and an early language intervention for very young children (Department for Education, 2021). In the US the Department of Education have provided Rescue Plan Funds to allow schools to address the many impacts of Covid‐19 on pre‐K to Grade 12 education (US Department of Education, 2021). A plethora of education‐related research has been, and continues to be, undertaken to better understand the impact of the pandemic on pupil and teacher outcomes, including the success or otherwise of remediation strategies and interventions.

### 1.2 Why it is important to develop the evidence and gap map (EGM)

The purpose of this EGM therefore is to create a repository of the educational research that has been conducted since the start of the pandemic in January 2020. Studies included in the map will be those that explore the effect of the pandemic, and subsequent remediation strategies, on pre‐primary, primary, secondary and special education school pupils' academic and wellbeing outcomes, as well as teacher outcomes. The map will be created using robust search, retrieval, and methodological approaches to minimise potential sources of research bias. It will be made publicly available and will provide a visual presentation of the educational research described above. The map will enable gaps in evidence to be identified as well as highlight areas in which there is sufficient research for evidence synthesis. The benefits are considerable, not only to pupil and teacher outcomes, but also: (1) funders can quickly assess the areas where there is already a saturation of evidence, see where there are gaps in knowledge, and direct much‐needed resources towards those areas; (2) Practitioners and policymakers can access the map to see where evidence exists to inform policy and practice; (3) Researchers can minimise research waste which occurs due to duplication of effort; and (4) Members of the public can quickly access information which is of relevance to them.

### 1.3 Existing EGMs and/or relevant systematic reviews

To our knowledge, no EGMs exist that collate the available education research evidence related to pupil and teacher outcomes as a consequence of the pandemic. A living systematic review on schools and emergency remote education for K‐12 during the COVID‐19 pandemic has been published and is being updated as new evidence emerges (Bond, 2021). However, our proposed map is broader in scope and includes a wider set of pupil outcomes, as well as teacher outcomes.

## 2 OBJECTIVES

Identify and map all existing primary studies, systematic reviews (published and unpublished), guidelines and policies on education during the Covid‐19 pandemic, creating a live, searchable and publicly available EGM.

## 3 METHODOLOGY

EGMs are a tool to prioritise research needs and to support evidence‐informed practice and policy decisions. The Campbell Collaboration methodological guidelines for EGMs will be adhered to (White et al., 2020) and the project will be conducted according to six stages:

(1) scoping and development of the EGM framework;
(2) systematic and comprehensive searches;
(3) screening for eligibility (i.e., title, then abstract, then full text);
(4) data extraction;
(5) high‐level quality appraisal of systematic reviews;
(6) and analysis (according to the predefined inclusion/exclusion criteria).

The first step of this EGM will be to develop the framework which best represents the research on education related to Covid‐19. The framework forms the basis for the systematic search, the screening and data extraction, and visual presentation of the included evidence.

### 3.1 Framework development and scope of the EGM

We will follow the standard EGM framework as a matrix, with rows containing the *type* of school and pupil that the research pertains to, that is, pre‐school/pre‐kindergarten, primary/kindergarten/elementary school, secondary/middle/high school, special school/education or multiple categories of school (where the setting, e.g., includes more than one category of school, such as pupils from both primary and secondary schools), and columns containing information regarding *outcomes* that is, teacher outcomes (physical, wellbeing, practices, attitudes) and child outcomes (attainment, physical, wellbeing, attitudes/behaviour). Guidelines and policy documents will also be mapped.

Additional information will also be coded by which the map can be filtered, including learning type (e.g., face to face, blended, online/virtual classroom), country of study, study design, mean age of children and whether the study reports an intervention or not.

The scope of this EGM has been driven by and informed by the Department for Education (DE) in Northern Ireland. Recognising the need for their decision making and guidance to schools to be based on the best available evidence from around the globe, DE commissioned the authors to undertake this map specifically to meet this need. The authors have also undertaken to identify, in collaboration with DE, areas from the map that are suitable for synthesis, and to create a number of evidence summaries. Various members of DE and the academic and education community will be engaged and consulted to inform each stage of the process.

### 3.2 Eligibility criteria

The inclusion and exclusion criteria were decided in consultation with all authors. Initial eligibility screening will be necessarily inclusive as our intention is to provide an overview of the body of evidence. Therefore, the team will review studies using the following eligibility questions:

1) Is the study focussed on Covid‐19 and its implications?
2) Are participants school aged children/young people and/or teachers?
3) Does the research have a specific focus on teaching and learning, an education setting and/or education‐related outcomes?

This eligibility criteria will be applied to each individual study at the title and abstract and we will include/omit based on relevance.

Studies that are focussed on preventing Covid‐19 infection and not on education outcomes will be excluded. Protocols of primary studies will also be excluded.

### 3.3 Dimensions

The EGM framework for an EGM informs the inclusion and exclusion criteria. We chose the *type* of school (and pupil) that the research pertains to, that is, pre‐school/pre‐kindergarten, primary/kindergarten/elementary school, secondary/middle/high school, special school/education multiple categories of school, and *outcomes*, that is, teacher outcomes (physical, wellbeing, teacher practices, attitudes) and child outcomes (attainment, physical, wellbeing, attitudes and/or behaviour) as our key dimensions. Adverse outcomes that fall into these outcome categories will also be coded.

#### 3.3.1 Types of study designs

We wish to identify all relevant primary studies and systematic reviews (published and unpublished). To capture this literature, we plan to include experimental and non‐experimental studies reported in scientific journal articles, preprints, book/book chapters, reports, and unpublished reports of education research conducted since the beginning of the Covid‐19 pandemic (January 2020) related to pupil and teacher outcomes. We also plan to include guidelines and policy documents that are captured by the searches as a separate category (column) in the map. These guidelines and policies will include those issued by national governments and education authorities. We will exclude guidelines and policies written for the ‘school level’.

Study designs which may be represented in the map include: Quantitative methods such as meta‐analysis, systematic review, randomised controlled trial, case‐control study, cohort study, cross sectional study, case reports and series. Qualitative methods such as systematic review, phenomenology, grounded theory, ethnography, historical, case study and mixed methods research. As Covid‐19 is still a novel disease and the implications on educational outcomes are not yet confirmed, it is important to be inclusive with study designs to get a fuller picture of the global body of evidence. We will exclude editorials, commentaries and opinion pieces.

We define both quantitative and qualitative systematic reviews as research which ‘seek to collate evidence that fits pre‐specified eligibility criteria in order to answer a specific research question. They aim to minimise bias by using explicit, systematic methods documented in advance with a protocol’. (Higgins et al., 2019). We define meta‐analysis as the statistical combination of results from two or more studies located through a systematic review.

#### 3.3.2 Population

We will include all pupils and teachers in pre‐school/pre‐kindergarten, primary/kindergarten/elementary school, secondary/middle/high school, and special school/education.

Special schools in the UK can specialise in one (or more) of four areas of special educational needs: communication and interaction; cognition and learning; social, emotional and mental health; and sensory and physical needs (https://www.gov.uk/types-of-school). In the US special education is defined as: ‘Specially designed instruction, at no cost to parents, to meet the unique needs of a child with a disability’ (Individuals with Disabilities Education Act, 2004).

#### 3.3.3 Context

We will include in the map all studies that report research in an educational context whether that learning context is face to face, virtual/online or a blended approach.

### 3.4 Search methods and sources

To ensure that the literature contained in the map is relevant and useful to key stakeholders, it is important that the literature retrieval methods follow high quality standards. Thus, the systematic search for literature will be conducted and reported by an information retrieval specialist (Author: C. K.) following Campbell Collaboration guidelines (White et al., 2020). Various literature sources will be searched, including electronic databases, web searches, conference proceedings, government reports and other repositories of literature. Only reports written in English (or with an English translation) will be included in the map.

### 3.5 Electronic databases

Based on the Queens's University Belfast database subscriptions, we will search the following key education databases:

- British Education Index (EBSCOhost)
- Child Development & Adolescent Studies (EBSCOhost)
- Educational Administration Abstracts (EBSCOhost)
- ERIC (EBSCOhost)
- International Bibliography of the Social Sciences (IBSS) (ProQuest)
- PsycINFO (1806–present) (Ovid)
- Scopus
- Social Science Citation Index (Web of Science Core Collection)

It will be necessary to adapt the search strategy for use in the various interfaces which host the listed databases, but an example search strategy for PsycINFO (1806–present; Ovid) is attached in Supporting Information: Appendix A.

### 3.6 Other sources

We will also search for grey literature across multiple sources. Grey literature is that which is not published, not peer reviewed, and not easily accessible. Sources of grey literature are varied and include government reports, privately and publicly funded research, conference proceedings, working papers, dissertations, and posters.

We will search for unpublished masters and doctoral dissertations on ProQuest Dissertations & Theses Global.

Google Scholar will be searched for additional forms of grey literature including government reports and working papers. Google Scholar limits the search to 256 characters and will export only the first 1000 records (Haddaway et al., 2015) which we will locate using the following search strategy:

(coronavirus|covid|SARS‐CoV‐2)(child*|pupil*|staff|student*|teacher*)(‘educational setting’|‘educational settings’|‘grade school’|‘high school’|kindergarten|school*)

We will search for relevant EGMs, systematic reviews and meta‐analyses via The Campbell Library, The Evidence for Policy and Practice Information and Co‐ordinating Centre (EPPI‐Centre), and The Social Care Institute for Excellence (SCIE). We will also search PROSPERO (University of York) for any protocols relevant to Education. We will handsearch the reference lists of all relevant systematic reviews to identify any eligible studies. A living systematic review on schools and emergency remote education for K‐12 during the COVID‐19 pandemic has been published and is being updated as new evidence emerges (Bond, 2021), this related review is more limited in scope, however, we will download the references from this map and deduplicate against our own references, to ensure that no relevant studies are missed.

It is expected that some studies located via the grey literature search will never be published or available in full. We will list these studies as ‘ongoing’ in the reference list of the final report. This will allow us to easily add these studies during map updates, if they are made available.

### 3.7 Screening and selection of studies

When all searches have been conducted, results will be imported to Endnote 20 where duplications of identical studies gathered from multiple sources can be removed to avoid duplication of effort.

### 3.8 Data extraction, coding and management

A data extraction tool has been developed by the authors and is provided in Appendix B. This will be piloted across 10% of included studies to ensure consistency and a high interrater reliability (assessed using *κ* statistic). Screening and data extraction will be undertaken in EPPI Reviewer software (Thomas et al., 2010). All studies will be screened initially by a single author, however, all included studies will be screened in duplicate independently. Ten percent of the data extraction will be undertaken in duplicate.

If multiple studies are reported in the same publication each separate study will be represented in the map separately. Equally, if there are multiple reports of a single study we will treat these as a single study.

### 3.9 Quality appraisal

The methodological quality of systematic reviews will be assessed using AMSTAR‐2 (Shea et al., 2017) and 10% will be assessed in duplicate.

## 4 ANALYSIS AND PRESENTATION

An interactive map using EPPI‐mapper software will be created and a test map will be piloted before making a final version available publicly online. The map will summarise all of the existing and emerging evidence in one place, for the first time. The results will be presented visually, clearly identifying where evidence exists, the nature of that evidence, and where there are gaps in the evidence base. The columns of the map will represent pupil and teacher outcomes, and the rows will represent the type, or stage, of schooling. Filters will allow users of the map to identify the country in which the study was conducted, the study design (including whether an intervention was studied), the learning context, and pupil mean age. The map will be accompanied by a descriptive report that will detail the EGM methodology, the main findings of the map, including additional findings through the use of filters, implications for policy and future research as well as a plain language summary.

## 5 STAKEHOLDER ENGAGEMENT

The EGM is being conducted for and in consultation with the Department of Education (DE), Northern Ireland, who is being engaged at each stage of the mapping process. Throughout the development of the EGM and the protocol, the author team met online at least monthly with a core team of three to four colleagues from DE to ensure that the conceptualisation of the map is appropriate to their purposes. In addition, at key stages of decision making (e.g., when defining the scope and eligibility criteria) the author team prepared summary documents to be circulated more widely within the DE, and through this third‐party mechanism, further feedback was provided via the core DE team to the author team.

This EGM is part of a wider piece of work that seeks to generate several evidence syntheses arising from the evidence contained within the EGM, specifically deigned to inform DE's planning and strategy in addressing the challenges arising from the pandemic in relation to education in Northern Ireland. Once the EGM is completed the author team plan to present the results of the map, and the evidence synthesis, via an internal webinar to the wider Department of Education.

## ROLES AND RESPONSIBILITIES

Content: Sarah Miller, Ciara Keenan, Erin Early
EGM methods: Sarah Miller, Ciara Keenan, Erin Early, Karen McConnell, Leonor Rodriguez
Information retrieval: Ciara Keenan, Leonor Rodriguez.

## SOURCES OF SUPPORT

This EGM is supported by the Department for Education, Northern Ireland.

## DECLARATIONS OF INTEREST

None to declare.

## PRELIMINARY TIMEFRAME

Approximate date for submission of the EGM: June 2022.

## PLANS FOR UPDATING THE EGM

This EGM will be updated every 2 years.

## Supporting information

Supporting information.
