# Supplementary material for: PROTOCOL: Education and Covid‐19: An evidence and gap map
Source: Campbell Syst Rev. 2023 Mar 19;19(1):e1318. doi: 10.1002/cl2.1318 (PMC10024917; doi:10.1002/cl2.1318)
Supplement: Supplementary file 1 — Supporting information. [file CL2-19-e1318-s001.docx]

## Appendix A: Search Strategy

## PsycINFO (1806 - present) (Ovid)

Pilot search conducted on 04 March 2021 at 15:49 GMT

| 1 | exp coronavirus/ | 2529 |
| --- | --- | --- |
| 2 | ((corona* or corono*) adj1 (virus* or viral* or virinae*)).ti,ab. | 41 |
| 3 | (coronavirus* or coronovirus* or coronavirinae* or Coronavirus* or Coronovirus* or Wuhan* or Hubei* or Huanan or "2019-nCoV" or 2019nCoV or nCoV2019 or "nCoV-2019" or "COVID-19" or COVID19 or "CORVID-19" or CORVID19 or "WN-CoV" or WNCoV or "HCoV-19" or HCoV19 or CoV or "2019 novel*" or Ncov or "n-cov" or "SARS-CoV-2" or "SARSCoV-2" or "SARSCoV2" or "SARS-CoV2" or SARSCov19 or "SARS-Cov19" or "SARSCov-19" or "SARS-Cov-19" or Ncovor or Ncorona* or Ncorono* or NcovWuhan* or NcovHubei* or NcovChina* or NcovChinese*).ti,ab. | 4571 |
| 4 | (((respiratory* adj2 (symptom* or disease* or illness* or condition*)) or "seafood market*" or "food market*") adj10 (Wuhan* or Hubei* or China* or Chinese* or Huanan*)).ti,ab. | 19 |
| 5 | ((outbreak* or wildlife* or pandemic* or epidemic*) adj1 (China* or Chinese* or Huanan*)).ti,ab. | 6 |
| 6 | "severe acute respiratory syndrome*".ti,ab. | 358 |
| 7 | or/1-6 | 4930 |
| 8 | exp Schools/ | 71140 |
| 9 | exp Education/ | 435783 |
| 10 | exp Curriculum/ | 120342 |
| 11 | exp Teaching/ | 124157 |
| 12 | exp Preschool Education/ | 4594 |
| 13 | exp Elementary schools/ | 8327 |
| 14 | exp Middle schools/ | 4311 |
| 15 | exp High schools/ | 7330 |
| 16 | educational setting$1.ti,ab. | 5237 |
| 17 | (school* or school?based or class* or educat* or curricul* or preschool* or pre?school* or kindergarten* or "primary school*" or "primary education" or pre?school* or elementary or post*primary or highschool* or high?school* or "junior high" or "middle school" or "middle education" or "technical college*" or "form college*").ti,ab. | 1058264 |
| 18 | (school* adj2 (pre or primary or secondary or high or middle or intermediate)).ti,ab. | 124692 |
| 19 | or/8-18 | 1194829 |
| 20 | exp Students/ | 270661 |
| 21 | exp Preschool Students/ | 12061 |
| 22 | exp Elementary School Students/ | 44373 |
| 23 | exp Middle School Students/ | 8972 |
| 24 | exp High School Students/ | 32585 |
| 25 | (Age*1 or "1 year* old*" or Age*2 or "2 year* old*" or Age*3 or "3 year* old*" or Age*4 or "4 year* old*" or Age*5 or "5 year* old*" or Age*6 or "6 year* old*" or Age*7 or "7 year* old*" or Age*8 or "8 year* old*" or Age*9 or "9 year* old*" or Age*10 or "10 year* old*" or Age*11 or "11 year* old*" or Age*12 or "12 year* old*" or Age*13 or "13 year* old*" or Age*14 or "14 year* old*" or Age*15 or "15 year* old*" or Age*16 or "16 year* old*" or Age*17 or "17 year* old*" or Age*18 or "18 year* old*").ti,ab. | 941425 |
| 26 | ("Grade* 1" or "Grade* 2" or "Grade* 3" or "Grade* 4" or "Grade* 5" or "Grade* 6" or "Grade* 7" or "Grade* 8" or "Grade* 9" or "Grade* 10" or "Grade* 11" or "Grade* 12" or "Grade* one" or "Grade* two" or "Grade* three" or "Grade* four" or "Grade* five" or "Grade* six" or "Grade* seven" or "Grade* eight" or "Grade* nine" or "Grade* ten" or "Grade* eleven" or "Grade* twelve" or "1st Grade*" or "2nd Grade*" or "3rd Grade*" or "4th Grade*" or "5th Grade*" or "6th Grade*" or "7th Grade*" or "8th Grade*" or "9th Grade*" or "10th Grade*" or "11th Grade*" or "12th Grade*" or "First Grade*" or "Second Grade*" or "Third Grade*" or "Fourth Grade*" or "Fifth Grade*" or "Sixth Grade*" or "seventh grade*" or "eight grade*" or "ninth grade*" or "tenth grade*" or "eleventh grade*" or "twelfth grade*").ti,ab. | 94517 |
| 27 | (child* or school?age* or youth* or pupil or adolesc* or young* or "peer-group" or peer* or sophomore or freshman or teen* or minor* or boy* or girl* or toddler* or infant* or junior*).ti,ab. | 1220367 |
| 28 | ("Junior infant*" or "1st year*" or "First Year*" or Year*1 or Year*2 or Year*3 or Year*4 or Year*5 or Year*6 or Year*7 or Year*8 or Year*9 or Year*10 or Year*11 or "Year*12 lower sixth" or "lower 6*OR Year*13 upper sixth" or "upper 6*").ti,ab. | 643025 |
| 29 | or/21-28 | 2008694 |
| **30** | **7 and 19 and 29** | **577** |

## Appendix B: Coding Framework

**Publication**

Article metadata [string]

**Possible Answers:** Authors; Year (published); Title; Journal name; DOI

Publication type [categorical]

**Possible Answers:** Journal Article; Preprint; Book/book chapter; Report; Unpublished report

**Outcomes**

Teacher outcomes: [categorical]

**Possible Answers:** Teacher physical; Teacher practices, Teacher wellbeing, Teacher attitudes

Pupil outcomes: [categorical]

**Possible Answers:** Pupil attainment, Pupil physical; Pupil attitudes/behaviour, Pupil wellbeing

Guidelines and policy document: [categorical]

**Possible answers:** Yes/no

**Context**

Type of school: [categorical]

**Possible Answers:** Pre-school, Primary school, Secondary school, Special school, Multiple

**Study design**

Quantitative methods: [categorical]

**Possible Answers:** Meta-analysis, Systematic review, Randomised controlled trial, Case-control study, Cohort study, Cross sectional study, Case reports and series

Qualitative methods: [categorical]

**Possible answers:** Systematic review, Phenomenology, Grounded theory, Ethnography, Historical, Case study

Mixed methods: [categorical]

**Possible answers:** yes/no

**Is this an intervention: [categorical]**

**Possible answers:** yes/no

**Mean age of children: [categorical]**

**Possible answers:** 0-3, 4-7, 8-11, 12-15, 16-18, none provided

**Learning mode: [categorical]**

**Possible Answers:** Face to face, Blended learning, online/virtual classroom, not clear

**Countries: [categorical]**

**Possible Answers:** All countries listed

**Notes [string]**

**Possible Answers:** [open]
